# Use of the experience sampling method in adolescents with Duchenne muscular dystrophy: a feasibility study

**DOI:** 10.1007/s00787-023-02317-2

**Published:** 2023-11-01

**Authors:** Judith M. Lionarons, Philippe A. E. G. Delespaul, Danique M. J. Hellebrekers, Martinus P. G. Broen, Sylvia Klinkenberg, Catharina G. Faber, Jos G. M. Hendriksen, Johan S. H. Vles

**Affiliations:** 1 School for Mental Health and Neuroscience, Maastricht University, Maastricht, The Netherlands; 2 Center for Neurological Learning Disabilities, Kempenhaeghe, Heeze, The Netherlands; 3 Department of Neurology, Maastricht University Medical Centre, Maastricht, The Netherlands; 4 Department of Adult Psychiatry, Mondriaan Mental Health Trust, Heerlen, The Netherlands; 5 Duchenne Centre Netherlands, Leiden, The Netherlands

**Keywords:** Duchenne muscular dystrophy, Steroid treatment, Cognition, Behaviour, Experience sampling method, mHealth

## Abstract

Experience sampling methods (ESM) using mobile health (mHealth) technology with a smartphone application are increasingly used in clinical practice and research. Still, recommendations are limited in young people, and adaptations may be necessary. Patients with Duchenne muscular dystrophy (DMD) are chronically treated with steroids from a young age. However, the impact of intermittent treatment schedules on fluctuations in somatic, cognitive and behavioural symptoms is poorly investigated. Existing studies are often cross-sectional and occur in controlled clinical settings, which do not provide sufficiently detailed insights into possible correlations. ESM might alleviate these problems. ESM innovates data collection with a smartphone application, which repeatedly assesses specific symptoms and contextual factors at random moments in daily life. We aimed to evaluate its feasibility in adolescents with DMD. In three (without/with/without steroids) 4-day periods of ESM, that were nested in 10/10 or 11/9 day on/off-medication periods, we evaluated its user-friendliness and compliance, and explored its ability to objectify fluctuations in somatic, cognitive and behavioural symptom severity and their relationship with contextual factors in seven DMD patients (age range 12–18 years) using intermittent corticosteroid treatment (dosage range 0.3–0.6 mg/kg/day). Patients reported that ESM was convenient and user-friendly. We were able to capture extensive intra-individual symptom fluctuations during intermittent corticosteroid treatment that were not revealed by routine clinical assessment. Implementing ESM to evaluate symptom fluctuation patterns in relation to treatment effects shows promise in adolescents with DMD. Optimization in further research is needed.

**Supplementary Information:**

The online version contains supplementary material available at 10.1007/s00787-023-02317-2.

## Introduction

Duchenne muscular dystrophy (DMD) is the most common fatal genetic disorder diagnosed in childhood [1]. Next to characteristic progressive muscle weakness, secondary somatic symptoms (i.e. pain, fatigue and sleep problems), as well as neurodevelopmental, behavioural and emotional symptoms are common [2–4]. Since there is no cure available, patients are symptomatically treated with corticosteroids before substantial physical decline (4–6 years old) [5, 6]. In DMD, favourable corticosteroid effects are slowing of muscle degeneration [7–9] and the most common adverse effects are excessive weight gain, cushingoid appearance and hirsutism [10]. Adverse cognitive and behavioural effects (difficulty to maintain concentration, poor memory, insomnia, agitation, depression, emotional lability and irritability) are less reported, but are often a reason to discontinue or switch treatment [11, 12]. Perceived neurodevelopmental side effects have been demonstrated to decline the longer corticosteroids were used [13]. To minimise adverse effects, ‘on/off’ treatment schemes (e.g. 10 days on/10 days off corticosteroids) are often used [14, 15]. This may lead to fluctuations in somatic, cognitive and behavioural functioning, which play an important role in quality of life [4, 16]. Conventional research methods are unlikely to provide sufficiently detailed insights into possible correlations with corticosteroid treatment. For example, a high discrepancy between retrospective questionnaires and actual experiences was found in patients with depression and anxiety [17]. Assessing subjects’ actual state several times a day during normal daily life is possible with the experience sampling method (ESM). Making use of mobile health (mHealth) technology with a smartphone application (app) [18], ESM provides information about symptom frequency and severity and context (place, company of others and activities) [19, 20]. ESM is used in patients with neurological disorders [21, 22], psychopathology [23–26], asthma [27], irritable bowel syndrome [28] and migraine [29]. This method can be implemented in children from 7 years old from various backgrounds [30]. However, there are challenges in young people, as it may be difficult for them to remain motivated and compliant with long or complex study protocols [30]. Current recommendations are limited, and protocol adaptations (e.g. fewer evaluations and shorter study time periods) may be necessary.

We aimed to assess the feasibility of ESM by exploring its ability to detect associations between somatic, cognitive, behavioural symptom fluctuations and contextual factors during periods with and without corticosteroid use in a sample of adolescent DMD patients.

## Methods

### Patients

Patients with DMD were recruited through their child neurologist (S.K.) or neuropsychologist (J.G.M.H.) at the Centre for Neurological Learning Disabilities (CNL) of Kempenhaeghe (Heeze, The Netherlands). Inclusion criteria were: (1) male, (2) age between 12 and 18 years old, (3) genetically confirmed diagnosis for DMD, (4) intermittent prednisone treatment, (5) indication of global intelligence on the basis of the Peabody Picture Vocabulary Test third version (PPVT-III) [31] with a cut-off intelligence quotient (IQ) > 85, (6) physical ability to independently use a personal smartphone (adequate arm/hand function) and (7) adequate proficiency in Dutch.

### Study design

For this first pilot study, we chose to use all available ESM data. In a total of three study periods, participants were assessed with ESM for three or four fixed days a week (Monday–Wednesday–Friday–Sunday) seven times a day. No adjustments were made to the personal corticosteroid schemes, which in some cases led to longer study periods. After inclusion, participants were briefed at home in presence of a legal representative, and the PsyMate™ app (www.psymate.eu) was installed on their smartphone. The app was autonomously used by the participant. Feedback on the use of the app was weekly asked by telephone. All ESM data were uploaded anonymously to a central database. Patient characteristics, i.e. age, *DMD* gene mutation, comorbid diagnoses (i.e. attention-deficit hyperactivity disorder [ADHD], autism spectrum disorders [ASD] and obsessive–compulsive disorders [32, 33]), wheelchair dependence, learning deficits, education (regular/special), corticosteroid dosage and psychopharmacology (i.e. stimulants, antipsychotics or antidepressants) were asked for in an interview. Clinical data was extracted from the electronic patient files of Kempenhaeghe, including PPVT-III as a commonly used estimator of general IQ and the Personal Adjustment, and Role Skills Scale-Third edition (PARS-III) for psychosocial adjustment (cut-off < 73 clinically significant psychosocial adjustment problems) [34]. Indication of global intelligence was based on the PPVT-III as a measure of receptive language, and not for performance intelligence. The PPVT-III is not comparable with a regular IQ test. This is a procedure used more often [35–37] especially in children with motor disabilities, because these children have a disadvantage on general IQ tasks due to points deduction on speed on amongst other the performance tasks which are part of the general IQ calculation. Moreover, the PPVT-III can be used as a pre-screening tool for global functioning in children to determine whether they are able to participate in self-report studies as an alternative for extensive neuropsychological testing, and deemed sufficient in this feasibility study [38].

The PPVT-III and the PARS-III have been administered as part of standard regular care. All patients included in the ESM sample seen at the outpatient clinic of CNL within 1 year of onset (T0) of the ESM study.

### The experience sampling method (ESM)

**Fig. 1:**
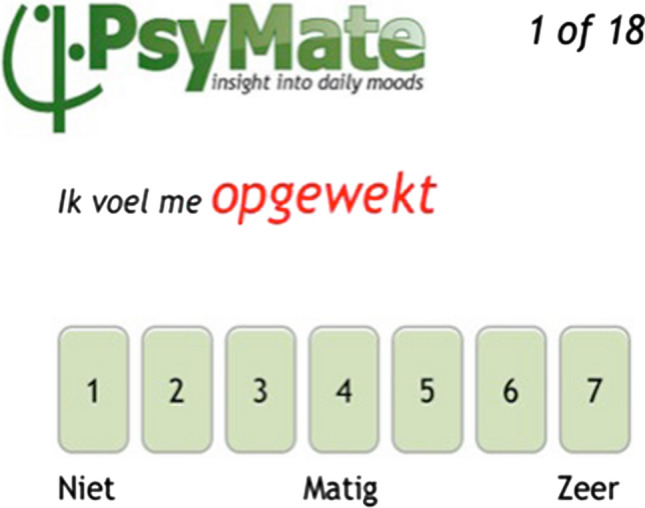
Experience sampling method question example. ‘I feel cheerful’ (in Dutch: ‘Ik voel me opgewekt’) on a 7-point Likert scale. A score of 1 indicated ‘not at all’ (in Dutch: ‘niet’), a score of 4 ‘moderate’ (in Dutch: ‘matig’) and a score of 7 ‘very much so’ (in Dutch: ‘zeer’)

To collect ESM data, the PsyMate™ app (www.psymate.eu) was customised for the present study: shorter total time window taking bedtime into account, < 10 evaluations a day, < 20 ESM questions per beep, assessments every other day during the week (during school) and in weekends, and relatively short study time periods (max. 4 days, every other day per study period). The app generated seven auditive beep signals (1:30–2:00 min/beep) per day at random moments with a minimal interval of 15 min and maximum of 120 min’ time blocks between 07.00 and 21.00 h, using a stratified random sampling (one beep by time block) for three or 4 days per medication condition: off corticosteroids (T0), on corticosteroids (T1) and off corticosteroids (T2) resulting in a total of 9–12 days with a total of 24–32 measurement points. They received 15 min to respond. Each questionnaire was designed to allow finishing within 2 min. At each beep, 18 questions (Fig. 1) were presented on the experience of: (1) somatic symptoms (fatigue, pain and sleep), (2) cognition (‘I am easily distracted’) and (3) mood (cheerful, satisfied, relaxed, insecure, lonely, irritated, guilty, down, frightened). An additional item ‘It is busy in my head’ was included, which has previously been used in ESM protocols [19], and is anecdotally reported by DMD patients and their parents. The items ‘I am easily distracted’ and ‘It is busy in my head’ were selected by three experts (i.e. neuropsychologist, child neurologist and psychiatrist) based on clinical experience and were put together in one domain for presentation purposes. All symptoms were rated by self-report on a 7-point Likert scale ranging from 1 = ‘not at all’ to 4 = ‘moderate’ to 7 = ‘very much so’. Contextual factors, such as the place (setting), company of others, and activities, were evaluated as well. The questions were the same for each measurement point. An overview of the questions is given in the Results section. Finally, participants were asked to fill out evaluative questions on sleep and overall experiences in a separate morning and evening questionnaire (Supplementary Tables S1 and S2), which took 1:00–1:30 min.

### Statistical analyses

PPVT-III and PARS-III scores were computed to compare the present DMD population with known DMD populations using one sample *t* tests [34, 35]. Missing items due to technical issues were excluded before ESM analysis.

Multi-level random regression techniques were identified as the most suitable method of data analysis of our ESM data. As previously described by Verhagen et al. [39], ESM observations are not independent (nested within subjects) and standard linear and logistic regression analysis techniques cannot be used. Dependent variables were mood, cognition, and somatic symptoms. Each question response on these variables was analysed using a separate regression model and there was a time component to each regression model. To optimise the estimation of the prediction variables (all modelled as fixed effects), we allowed between subject variation in intercept (random slopes). Whilst more sophisticated modelling is possible, we used this traditional approach considering the small sample size and the fact that the data analysis is part of a feasibility study.

Mood, cognition and somatic symptom fluctuations were assessed in relation to ‘on/off’ corticosteroid conditions in an A − B − A design (A = T0 and T2 [‘off’] and B = T1 [‘on’]). The A (‘off corticosteroids’) condition applied to both T0 and T2 and the data collected at both study time periods were considered under similar conditions, since the data were collected in subjects under stable medication regimes for at least 6 years. Both first and second ‘A’ conditions (T0 and T2) contain similar carry-over effects of a previous corticosteroid intervention (measured under ‘B’ [T1], but also present in the not-assessed T1 period).

Notably, the 7-point Likert scale responses are often not normally distributed in ESM. However, when transforming data to normalise the distribution, statistical differences change the distribution of the data, herewith inducing systematic and not wanted alterations of the empirically collected data (transformations are not neutral). Changing the data in this way potentially would have a great impact on the statistical outcomes. Therefore, we chose not to do this.

To assess the potential influence of contextual factors, we added interactions (with ‘alone/not alone’ and ‘at home/not at home’) in the multilevel random regression model. Each contextual factor was analysed in a separate model. Furthermore, sleep pattern and quality of sleep were assessed in relation to corticosteroid condition using a multilevel random regression model.

The fit-of-all-multilevel random regression models were indicated by Wald chi-squared tests for each individual predictor. All models were considered significant if *p* < 0.05. Since all variables were not normally distributed correlations were computed with Spearman’s rho. Strength of the correlation between the observed and modelled data was defined as *r* < 0.3 very small, *r* = 0.3–0.5 small, *r* = 0.5–0.7 moderate and *r* > 0.7 strong [40]. Since modelled data refer to the overall model, there can only be one rho by model. A different model was fitted for each mental state. Analyses were performed using the statistical software programme STATA (version 13.1 for Mac).

## Results

### Patients

Eight adolescents with DMD (age range = 12–18 years) participated in this study (Table 1). Six participants were treated with a 10 days on–10 days off prednisone treatment scheme; two participants switched in 11 days on–9 days off schedules. A maximum dosage of 30 mg/day was prescribed [5]. In some cases, the PPVT-III was not completed resulting in missing IQ scores (*n* = 3). Mean IQ estimation based on the PPVT-III score (*n* = 5) was 117.00 (standard deviation [SD] 16.20, range 92–135), which is higher than the general DMD population [98.15 (SD 20.24)] [35]. This difference was not statistically significant (*p* = 0.060) within our sample size but is clinically significant. The following comorbid diagnoses were present: ASD (*n* = 2) and ADHD (*n* = 1). Two participants had dyscalculia. Other neurodevelopmental and learning problems without clinical diagnoses were related to attention (*n* = 2), working memory (*n* = 1) and reading and spelling (*n* = 1). Total PARS-III score (*n* = 8) was on average above the clinical cut-off point (> 73) and significantly higher than in previous literature (89.38 vs. 84.43; *p* = 0.043) [34].

**Table 1:** Patient characteristics

Characteristic		
Participants, *n*	8	
Age, yr, mean (SD)	15.10 (1.90)	Range 12–18 yr
*DMD* mutations, *n*		
Exon 1–44 (Dp140+)^1^	4	
Exon 50–62 (Dp140−)^2^	4	
Prednisone treatment scheme, *n*		
10 days on–10 days off	6	
11 days on–9 days on	2	
Prednisone dose range, mg/kg/day	0.30–0.60	Max. dosage: 30 mg/day
Wheelchair bound, *n*	7	
Regular education, *n*	4	
Comorbid neuropsychiatric, neurodevelopmental or learning disorders, *n*		
ADHD	1	
ASD	2	
Dyscalculia	2	
PPVT-III IQ score, mean (SD)	117.00 (16.20)	
PPVT-III IQ range	92–135	
PARS-III, mean (SD)	89.38 (5.68)	

ADHD, Attention-deficit hyperactivity disorders; ASD, autism spectrum disorders; DMD, Duchenne muscular dystrophy; PARS-III, Personal Adjustment and Role Skills Scale-Third Edition for psychosocial adjustment; PPD-NOS, pervasive developmental disorder-not otherwise specified; PPVT-III IQ, Peabody Picture Vocabulary Test-Third edition for intelligence quotient (IQ) estimation

The following DMD mutations were classified: (1) mutations upstream of intron 44 were considered Dp140 + (intact Dp140) and (2) mutations involving the transcription start site in intron 44 or involving the genomic region downstream of exon 50 were considered Dp140-, as previously described [37]. Not all information was available for all variables for all patients.

### Compliance

Participants received a total of 72–96 continuous questionnaires, 9–12 morning questionnaires and 9–12 evening questionnaires. The continuous questionnaires were completed in (T0) 45%, (T1) 34% and (T2) 20% of measurement points. Morning and evening questionnaires were completed in (T0) 44% and 47%, (T1) 32% and 31% (T1) and (T2) 24% and 22% of measurement points. Six participants filled out less than one-third of beeps, which compromises ESM data validity [19]. One of these six participants prematurely terminated his participation after T1 due to technical problems, which reduced our sample size to 7 patients for further analysis. After resolving the technical problem, further participation was reported as being too burdensome.

### User-friendliness, evaluation and feedback

All participants found the information about the app clear. None of them changed their daily behaviour to fill out the ESM. Occasionally, beeps were missed when participants were in gym class or taking a school exam for example. All participants used their personal smartphone and found the app easy to use. Most participants (75%) did not mind that the continuous questionnaires showed up at random moments during the day and reported that filling out the app did not influence their mood.

### Ability to detect diurnal fluctuations in somatic, cognitive and behavioural symptoms in relation to contextual factors during intermittent corticosteroid treatment

**Fig. 2:**
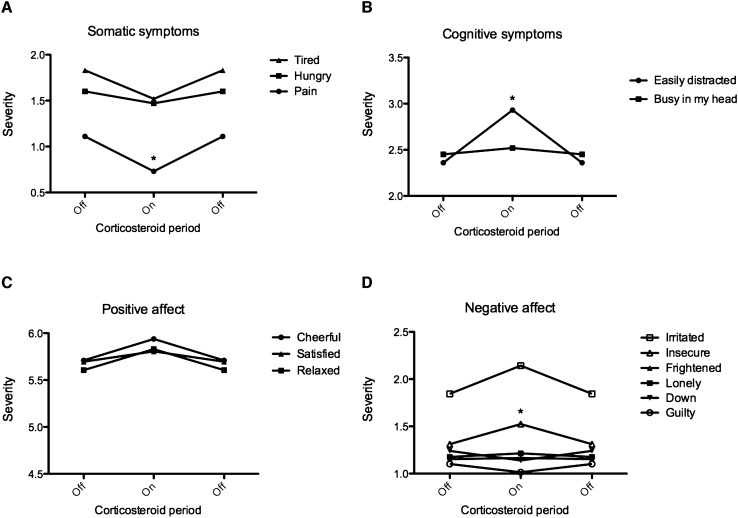
Visualisation of symptom fluctuations in relation to corticosteroid treatment. Data are presented as estimated severity per corticosteroid period in an A − B − A design (A = T0 and B = T1) based on a multilevel random regression model. *p < 0.05 (pain, easily distracted, insecure). *n* = 7. Off, study period without corticosteroids (T0 and T2); On, study period with corticosteroids (T1)

Descriptive statistics are shown in Table S3. ESM analysis (Table 2 and Fig. 2) showed a decrease in pain on corticosteroids. For fatigue and hungriness, no differences were found. Cognitive symptom analyses showed an increase in distraction on corticosteroids and no differences for ‘I feel busy in my head’. For cheerfulness, relaxation and satisfaction, no differences were found. An increase in insecurity was found on corticosteroids. For irritability, loneliness, fear, feeling down and guilty, no differences were found between the “on” and “off” corticosteroid treatment condition. The strength of the three significant correlations was very small or small (*r* = 0.02–0.46).

**Table 2:** Multilevel random regression analysis of somatic, cognitive and affective symptoms in relation to corticosteroid treatment

	Off	On	Off	Wald chi-square	*P*-value	Spearman’s rho
Item						
Somatic domain						
I am hungry	1.60	1.47	1.60	0.46	0.50	0
I am tired	1.83	1.52	1.83	3.08	0.08	0.25
I am in pain	1.11	0.73	1.11	10.01	0.002*	0.17
Cognitive domain						
I feel busy in my head	2.45	2.52	2.45	0.24	0.62	0.64
I am easily distracted	2.36	2.93	2.36	10.83	0.001*	0.46
Behavioural domain/affect						
I feel cheerful	5.71	5.94	5.71	2.03	0.15	0.31
I feel insecure	1.31	1.52	1.31	5.25	0.02*	0.02
I feel relaxed	5.61	5.83	5.61	1.79	0.18	0.22
I feel irritated	1.84	2.14	1.84	3.70	0.05	0.28
I feel satisfied	5.70	5.81	5.70	0.41	0.52	0.24
I feel lonely	1.18	1.21	1.18	0.29	0.59	0.12
I feel frightened	1.53	1.17	1.53	0.07	0.79	0.06
I feel down	1.24	1.14	1.24	1.42	0.23	0.15
I feel guilty	1.10	1.01	1.10	1.37	0.24	0.27

Data are presented as estimated severity per corticosteroid period based on a multilevel random regression model with Wald chi-square for model fit and Spearman’s rho for correlation strength

Off, study period without corticosteroids; On, study period with corticosteroids

*p < 0.05; *n* = 7

Additionally, participants handled both contextual conditions (‘alone-not alone’ and ‘at home-not at home’) differently between corticosteroid periods when looking into pain, fatigue, distraction and insecurity (Table S4). For hungriness, an interaction was only found between ‘at home-not at home’ and corticosteroid treatment condition. The strength of the correlations was very small to moderate (*r* = 0.01–0.54). For the remaining symptoms, no interaction effects were found.

### Ability to detect the effects of intermittent corticosteroid treatment on sleep pattern and quality of sleep

No relations were found between corticosteroid treatment conditions and time it took to fall asleep (Wald chi-square (1) = 0.12, n.s., *r* = 0), number of times of waking up during the night (Wald chi-square (1) = 1.77, n.s., *r* = 0.17) and time lying awake before getting up in the morning (Wald chi-square (1) = 1.74, n.s., *r* = 0). No relations were found between corticosteroid treatment conditions and quality of sleep (Wald chi-square (1) = 0.38, n.s., *r* = 0.24).

### Ability to detect intra-individual corticosteroid treatment effects

We expected a strong correlation between distractions levels and corticosteroid periods for the 14-year-old participant with a comorbid ADHD diagnosis. This participant was treated with 15 mg short-acting methylphenidate in the morning and 10 mg at noon. The participant was more easily distracted on corticosteroids [*F*(1, 34) = 5.21, *p* < 0.05, *R*^2^ = 0.13]. A similar pattern was found in distraction fluctuations for both corticosteroid conditions: in the morning when getting up and getting ready for school distraction levels increased; at school distraction levels first dropped and then increased in the afternoon to drop again before bedtime. When asked for, the participant reported he never used the second methylphenidate dose during this study.

## Discussion

The main finding of this pilot study is that ESM is a potentially applicable method in adolescent DMD patients and can detect intra-individual symptom fluctuations during intermittent corticosteroid treatment. The context of being alone or at home plays an important role in the experience of symptoms and differs whether participants are on corticosteroids.

### Feasibility of the ESM

Participants reported no disturbance of normal daily activities or mood. ESM was evaluated as convenient and user-friendly, and the overall experience was positive. However, compliance rates were low and decreased with successive time periods. One of eight participants prematurely terminated his participation after T1 due to technical problems, which reduced our sample size to seven patients for further analysis.

ESM completion rates in a large meta-analysis of chronic pain studies (*n* = 10 studies, *n* = 701 patients) found that younger respondents showed lower completion rates than older respondents, and showed a decline over the course of the studies [41]. Although the age in this meta-analysis ranged between 19 and 80 years, this is still valuable information and may suggest that the young age of our sample and use of multiple study periods (possible exhaustion and attrition bias) could potentially have affected completion rates and herewith statistical power. In addition, a meta-analysis on ESM compliance and retention in mental disorders showed better compliance when intervals between successive evaluation periods were longer and with fewer evaluations a day [42]. Because this study included relatively short intervals between periods also during school, this might have affected completion rates too. The number of evaluations was already relatively low in this study and should preferably not drop below six evaluations a day to reliably assess diurnal fluctuations. Nevertheless, ESM proved to be sensitive to capture extensive fluctuations in symptom severity and contextual factors during intermittent corticosteroid treatment. Also, it is important to mention that the current study used a convenience sample to test the feasibility of ESM in a sample of adolescents with DMD (mean age 15.10 years) with sufficient smartphone skills and cognitive abilities to complete the whole ESM trajectory. This bias may have influenced the data and may suggest that ESM is only feasible in adolescents with a relatively high IQ. Therefore, future research in a lager sample with a more representative IQ range is necessary. As mentioned in the *Study design* section, the PVVT-III was used as more of a pre-screening tool for study entry. To compare IQ more reliably between our sample and general DMD population, extensive neuropsychological testing is necessary.

### Measuring intermittent corticosteroid treatment effects with the ESM

Our findings support previous literature on decreased pain and increased levels of distraction and insecurity in relation to corticosteroid use [11, 43]. The observed fluctuations throughout the day in the intra-individual analysis of the participant with a comorbid ADHD diagnosis may suggest a strong link with the methylphenidate dosage scheme used, since the afternoon dose was not used during this study. In clinics, this could possibly be an argument to adjust the methylphenidate dosage scheme to ‘on/off’ periods of corticosteroids. No other comorbid diagnoses were investigated, because in the case for ADHD, there is a clear overlap in ADHD symptoms and adverse effects of corticosteroids, which is not the case for ASS or dyscalculia.

Although sleep disorders have been reported as an adverse effect of corticosteroids [11], no relations were found between on/off corticosteroid treatment conditions and sleep pattern or quality. This suggests that the relatively low corticosteroid dosages (0.3–0.6 mg/kg/day) might have less impact on sleep than on the other investigated symptoms.

### Contextualization of the ESM in relation to intermittent corticosteroid treatment

Some of the contextual effects found, may potentially be artefacts of the daily structure of the participants; for hungriness and fatigue, it is logical to experience these complaints when being at home. Also, young and wheelchair-dependent people between 12 and 18 years old are most often not alone when they are at home. Since avoidance is common behaviour in patients with chronic pain [44], behavioural selection may play a role in this: when in pain, people are less likely to go outside. When experiencing cognitive symptoms (being distracted), people are less likely to go outside and/or engage in social activities. Previous research in schizophrenia and depression, demonstrated that cognitive deficits contribute to poor psychosocial functioning [45, 46], we observed a similar pattern in our DMD sample. Finally, being not at home is a less familiar/unknown situation, possibly around people they are less close to than family (i.e. classmates), which could result in the experience of insecurity.

### Limitations

For ESM, a certain level of fine-motor function is required to use a smartphone. This may be a problem in older DMD patients with progressed muscle dysfunction of the upper limbs [47]. It is important to note that ESM requires considerable motivation of the patient, which should be asked for in an interview beforehand to enhance compliance rates [48]. This is particularly important in a small sample size, which is often the case in pilot studies. On top of this, a lack of motivation could potentially impact the analysis and make it more difficult to translate the conclusions of the study to the general DMD population.

Furthermore, participants were referred to the outpatient clinic of the Centre for Neurological Learning Disabilities of Kempenhaeghe because of possible comorbid problems in learning and behaviour, which could potentially be a bias. In the current study, our sample experienced more psychosocial adjustment problems based on the PARS-III compared with the general DMD population. However, as previously reported by Hendriksen et al. [34], we would also not expect that the presence of psychosocial adjustment problems is influenced by steroid use in the current study. Especially given the small sample in the current study and the relatively older age (only adolescents), in the current study, the sample is too small to speculate about these relations. Although our model limits the conclusions that can be drawn from this finding, there was no indication that steroids played a major role in negatively modifying psychosocial adjustment in our sample.

Finally, participants had to login and out of the app for each study period, which led to problems in one participant. As the study results were analysed in A–B–A model, it was unlikely that this had influenced our results. Nonetheless, complex and repeated logins should be avoided. However, on the other hand, the A–B–A model also has its limitations; assuming that both ‘off’ corticosteroid periods (T0 and T2) are similar, does not take all possible confounding factors that could have influenced the outcome into account (i.e. motivation).

### Clinical implications

ESM can capture extensive symptom fluctuations during intermittent corticosteroid treatment. Based on clinical observations, these effects were not revealed by routine clinical assessment. However, this should be further investigated by comparing both methods in a follow-up study. This method avoids methodological problems of retrospective questionnaires or diaries, such as recall bias or ‘back-filling’. ESM facilitates the recognition of individual patterns in treatment reactivity. By this, it enhances understanding of symptom severity variation and may facilitate treatment optimization, as well as self-management [49]. ESM can be used from a young age (≥ 7 years old [30]). We showed that adolescents with DMD are capable to autonomously use ESM. In older DMD patients, speech-driven technology may be of help to avoid the motor function requirement, which should be a topic of future research. Also, ESM could predict important transitions over the course of progressive diseases, which can be an interesting topic of future studies. Symptom variability can be related to contextual factors using ESM, allowing to better understand stress-related vulnerabilities [50, 51]. More specific and extensive cognitive ESM assessments should be further investigated compared to regular neuropsychological evaluation. Recent ESM developments allow assessments of within-day cognitive performance [52]. Furthermore, the ability of ESM to assess multiple repeated measurements within individual subjects makes single subject predictions possible [49], thus making ESM suitable for scientific research on rare diseases with a relatively small sample size. Based on our findings and previous research, it is recommended to increase intervals between successive evaluation periods for children and adolescents [42]. Another strategy to enhance compliance in young people could be to include elements of ‘gamification’ to motivate participants [48].

## Conclusions

ESM is a feasible method in our DMD sample of adolescents. It may reveal potential pattern alterations in relation to treatment, which allows better targeted and personalised interventions or pharmacotherapy. Still, optimization and compliance enhancement in young people is needed in a larger sample with a broader IQ range based on more extensive neuropsychological testing.

### Supplementary Information

Below is the link to the electronic supplementary material.

Supplementary file 1 (DOCX 29 kb)

## Data Availability

As there is a possibility to identify participants based on their clinical and experience sampling data, the datasets generated and/or analyzed during the current study cannot be made publicly available based on European law.
