# Supplementary material for: Use of the experience sampling method in adolescents with Duchenne muscular dystrophy: a feasibility study
Source: Eur Child Adolesc Psychiatry. 2023 Nov 1;33(7):2281–90. doi: 10.1007/s00787-023-02317-2 (PMC11255059; doi:10.1007/s00787-023-02317-2)
Supplement: Supplementary file 1 — (DOCX 29 kb) [file 787_2023_2317_MOESM1_ESM.docx]

**Supporting information**

**Supplementary table 1. Morning questionnaire.**

| **Item** | **Scale** |
| --- | --- |
| How long did it take before I fell asleep yesterday evening? | 0-5 min, 5-15 min, 15-30 min, 30-45 min, 45 min-1 h, 1-2 h, 2-4 h, > 4 h |
| How often did I wake up last night? | 0, 1, 2, 3, 4, 5, >5 |
| How long did I lie awake before getting up this morning? | 0-5 min, 5-15 min, 15-30 min, 30-45 min, 45 min-1 h, 1-2 h, 2-4 h, > 4 h |
| I slept well | 1= not at all 4 = moderate 7 = very much so |

**Supplementary table 2. Evening questionnaire.**

| **Item** | **Scale** |
| --- | --- |
| Overall I felt good today | 1 = not at all 4 = moderate 7 = very much so |
| Overall I felt tired today | 1 = not at all 4 = moderate 7 = very much so |
| Overall I felt tense today | 1 = not at all 4 = moderate 7 = very much so |
| Filling out the PsyMate has influenced my mood | 1 = not at all 4 = moderate 7 = very much so |
| Without the PsyMate I would have done something else today | 1 = not at all 4 = moderate 7 = very much so |

**Supplementary table 3. Overview of all study variables for the three time periods.**

|  | **T0** | **T1** | **T2** | **Overall** |
| --- | --- | --- | --- | --- |
| **Item** |  |  |  |  |
| *Somatic domain^a^*  I am hungry  I am tired  I am in pain | 1.80 (1.57)  1.95 (1.36)  1.33 (0.87) | 1.60 (1.34)  1.80 (1.26)  1.03 (0.18) | 1.61 (1.39)  2.46 (1.91)  1.70 (1.64) | 1.69 (1.45)  2.00 (1.48)  1.31 (0.98) |
| *Cognitive domain^a^*  I feel busy in my head  I am easily distracted | 2.55 (1.91)  1.99 (1.67) | 1.93 (1.92)  2.24 (2.04) | 2.35 (2.17)  2.04 (1.80) | 2.40 (1.93)  2.02 (1.77) |
| *Behavioral domain/affect^a^*  I feel cheerful  I feel insecure  I feel relaxed  I feel irritated  I feel satisfied  I feel lonely  I feel frightened  I feel down  I feel guilty | 5.08 (1.49)  1.24 (0.72)  5.25 (1.47)  1.41 (0.97)  5.37 (1.61)  1.16 (0.58)  1.16 (0.38)  1.34 (0.80)  1.17 (0.57) | 5.92 (1.13)  1.17 (1.00)  5.89 (1.32)  1.76 (1.54)  5.96 (1.28)  1.10 (0.64)  1.08 (0.37)  1.10 (0.45)  1.09 (0.44) | 5.80 (1.35)  1.00 (0.00)  5.45 (1.44)  2.02 (1.84)  5.61 (1.41)  1.08 (0.40)  1.10 (0.51)  1.20 (0.76)  1.31 (1.10) | 5.47 (1.39)  1.16 (0.73)  5.51 (1.41)  1.59 (1.33)  5.62 (1.47)  1.12 (0.55)  1.12 (0.40)  1.25 (0.71)  1.16 (0.66) |
| *Context*  What am I doing, %  Work/school  Housekeeping  Hygiene  Relaxation, passive  Relaxation, active  Sports  Eating/drinking  Social interaction, offline  Social interaction, online  Something else  Would I preferably do something else^a^  Where am I, %  At home  At some else’s home  At school/work  At a public place  On the go  Somewhere else  With who am I, %.  No one  Partner  Family living in  Family away from home  Friends  Colleagues  Acquaintance  Stranger | 33.33  0.00  5.71  18.10  12.38  0.95  13.33  0.95  8.57  6.67  2.65 (2.28)  62.90  0  30.48  3.81  3.81  0  16.19  0  36.19  1.90  36.19  4.76  3.81  0.95 | 24.18  1.10  2.20  15.38  8.79  1.10  19.78  3.30  4.40  19.78  2.49 (2.09)  53.85  0  18.68  6.59  5.49  15.38  7.78  0  62.22  0  24.44  3.33  1.11  1.11 | 31.48  0.00  3.70  29.63  14.81  0.00  3.70  0.00  3.70  12.96  2.61 (2.24)  59.26  3.70  18.52  1.85  7.41  9.26  18.52  0  48.15  0  24.07  3.70  3.70  1.85 | 29.60  0.40  4.00  19.60  11.60  0.80  13.60  1.60  6.00  12.80  2.58 (2.20)  58.40  0.80  23.60  4.40  5.20  7.60  13.65  0  48.19  0.8  29.32  4.02  2.81  1.20 |

^a^Data are presented as mean with standard deviation of a 7-point Likert scale (0-7). N= 7.

T0 = first study period without corticosteroids; T1 = second study period with corticosteroids; T2 = third study period without corticosteroids.

**Supplementary table 4. Multilevel random regression analysis of context in relation to corticosteroid treatment.**

|  | **Off** | **On** | **Off** | **Wald chi-square** | **P-value** | **Spearman’s rho** |
| --- | --- | --- | --- | --- | --- | --- |
| **Item** |  |  |  |  |  |  |
| *Somatic domain^a^*  I am hungry  Alone  Not alone  At home  Not at home  I am tired  Alone  Not alone  At home  Not at home  I am in pain  Alone  Not alone  At home  Not at home | 1.00  1.66  1.31  0.92  2.30  1.80  2.05  1.72  1.30  1.08  1.30  0.91 | 0.71  1.50  1.09  0.40  1.93  1.55  1.73  1.56  1.00  0.70  1.07  0.39 | 1.00  1.66  1.31  0.92  2.30  1.80  2.05  1.72  1.30  1.08  1.30  0.91 | 4.78  13.97  8.60  8.85  10.72  13.97 | .19  .003*  .04*  .03*  .01*  .003* | 0.00  0.16  0.25  0.30  0.22  0.16 |
| *Cognitive domain^a^*  I feel busy in my head  Alone  Not alone  At home  Not at home  I am easily distracted  Alone  Not alone  At home  Not at home | 2.11  2.36  2.13  2.48  1.80  2.50  2.37  2.51 | 3.18  2.80  2.67  2.04  1.51  2.57  2.44  2.53 | 2.11  2.36  2.13  2.48  1.80  2.50  2.37  2.51 | 20.43  15.55  4.43  1.41 | <.001*  .001*  .22  .70 | 0.39  0.54  0.59  0.71 |
| *Behavioral domain/affect^a^*  I feel cheerful  Alone  Not alone  At home  Not at home  I feel insecure  Alone  Not alone  At home  Not at home  I feel relaxed  Alone  Not alone  At home  Not at home  I feel irritated  Alone  Not alone  At home  Not at home  I feel satisfied  Alone  Not alone  At home  Not at home  I feel lonely  Alone  Not alone  At home  Not at home  I feel frightened  Alone  Not alone  At home  Not at home  I feel down  Alone  Not alone  At home  Not at home  I feel guilty  Alone  Not alone  At home  Not at home | 5.59  5.73  5.66  5.78  0.97  1.33  1.04  1.61  5.19  5.63  5.76  5.57  2.20  1.81  1.77  2.01  5.33  5.69  5.72  5.66  1.04  1.18  1.19  1.08  1.02  1.17  1.09  1.16  1.29  1.24  1.27  1.20  1.16  1.10  1.10  1.06 | 5.27  6.04  5.91  6.04  0.98  1.54  1.05  2.03  4.51  5.97  5.63  6.39  1.48  2.04  1.84  2.63  5.34  5.78  5.87  5.72  1.00  1.21  1.29  0.96  1.01  1.18  1.09  1.11  1.10  1.15  1.23  1.02  1.18  1.00  1.07  0.88 | 5.59  5.73  5.66  5.78  0.97  1.33  1.04  1.61  5.19  5.63  5.76  5.57  2.20  1.81  1.77  2.01  5.33  5.69  5.72  5.66  1.04  1.18  1.19  1.08  1.02  1.17  1.09  1.16  1.29  1.24  1.27  1.20  1.16  1.10  1.10  1.06 | 5.40  3.12  7.95  22.61  6.81  5.71  5.31  7.30  1.78  0.50  1.06  2.72  3.64  4.71  2.74  2.09  1.65  2.80 | .14  .37  .047*  <.001*  .08  .13  .15  .06  .62  .92  .79  .44  .30  .19  .43  .55  .65  .42 | 0.38  0.11  0.02  0.01  0.29  0.05  0.28  0.27  0.24  0.23  0.09  0.03  0.08  0.01  0.13  0.16  0.22  0.34 |

Data are presented as estimated severity per corticosteroid period based on a multilevel random regression model with Wald chi-square for model fit and Spearman’s rho for correlation strength in the context of ‘alone-not alone’ and ‘at home-not at home’.

*p < .05. N = 7.

Off = study period without corticosteroids; On = study period with corticosteroids.
